# Comparative analysis of alignment algorithms for macular optical coherence tomography imaging

**DOI:** 10.1186/s40942-023-00497-2

**Published:** 2023-10-02

**Authors:** Craig K. Jones, Bochong Li, Jo-Hsuan Wu, Toshiya Nakaguchi, Ping Xuan, T. Y. Alvin Liu

**Affiliations:** 1 Wilmer Eye Institute, School of Medicine, Johns Hopkins University, 600 N. Wolfe Street, Baltimore, MD 21287 USA; 2 The Malone Center for Engineering in Healthcare, Johns Hopkins University, Malone Hall, Suite 340, 3400 North Charles Street, Baltimore, MD 21218 USA; 3 Graduate School of Science and Technology, Chiba University, 1-33, Yayoicho, Inage Ward, Chiba-shi, Chiba, 263-8522 Japan; 4 Shiley Eye Institute and Viterbi Family Department of Ophthalmology, University of California San Diego, 9415 Campus Point Drive, La Jolla, CA 92093 USA; 5 Center for Frontier Medical Engineering, Chiba University, 1-33, Yayoicho, Inage Ward, Chiba-shi, Chiba, 263-8522 Japan; 6 School of Computer Science and Technology, Heilongjiang University, Harbin, 150080 China

**Keywords:** Optical coherence tomography, B-scans, Image registration, Image alignment, Age-related macular degeneration

## Abstract

**Background:**

Optical coherence tomography (OCT) is the most important and commonly utilized imaging modality in ophthalmology and is especially crucial for the diagnosis and management of macular diseases. Each OCT volume is typically only available as a series of cross-sectional images (B-scans) that are accessible through proprietary software programs which accompany the OCT machines. To maximize the potential of OCT imaging for machine learning purposes, each OCT image should be analyzed en bloc as a 3D volume, which requires aligning all the cross-sectional images within a particular volume.

**Methods:**

A dataset of OCT B-scans obtained from 48 age-related macular degeneration (AMD) patients and 50 normal controls was used to evaluate five registration algorithms. After alignment of B-scans from each patient, an en face surface map was created to measure the registration quality, based on an automatically generated Laplace difference of the surface map–the smoother the surface map, the smaller the average Laplace difference. To demonstrate the usefulness of B-scan alignment, we trained a 3D convolutional neural network (CNN) to detect age-related macular degeneration (AMD) on OCT images and compared the performance of the model with and without B-scan alignment.

**Results:**

The mean Laplace difference of the surface map before registration was 27 ± 4.2 pixels for the AMD group and 26.6 ± 4 pixels for the control group. After alignment, the smoothness of the surface map was improved, with a mean Laplace difference of 5.5 ± 2.7 pixels for Advanced Normalization Tools Symmetric image Normalization (ANTs-SyN) registration algorithm in the AMD group and a mean Laplace difference of 4.3 ± 1.4.2 pixels for ANTs in the control group. Our 3D CNN achieved superior performance in detecting AMD, when aligned OCT B-scans were used (AUC 0.95 aligned vs. 0.89 unaligned).

**Conclusions:**

We introduced a novel metric to quantify OCT B-scan alignment and compared the effectiveness of five alignment algorithms. We confirmed that alignment could be improved in a statistically significant manner with readily available alignment algorithms that are available to the public, and the ANTs algorithm provided the most robust performance overall. We further demonstrated that alignment of OCT B-scans will likely be useful for training 3D CNN models.

## Background

### OCT background

Optical Coherence Tomography (OCT) is a major technological breakthrough in medical imaging, after the invention of computed tomography (CT) [1] and magnetic resonance imaging (MRI) [2]. OCT [3] has revolutionized the field of ophthalmology, as it can image tissue non-invasively at a micron level resolution, both for the anterior segment of the eye [4] and posterior segment of the eye [5]. It takes advantage of the different reflectivity of tissues to determine the delay time and reflection intensity of the emitted light waves, by comparing the reflected and reference light waves through a low coherence optical interferometer [6].

Within ophthalmology, OCT is particularly important for the diagnosis and management of different retinal diseases, for example age-related macular degeneration (AMD), which is the leading cause of central vision loss in persons over the age of 50 in the United States [7]. During the acquisition of an OCT image in a patient with AMD or other macular diseases, the scan area is centered on the macula, which is responsible for central high-resolution vision, and light rays are passed through the macula in an anterior–posterior fashion, producing numerous A-scans. After the image acquisition is completed, the A-scans are combined to create a single B-scan, which is a cross-sectional image of the macula. Within a scan area, multiple B-scans are typically captured, though the density of the B-scans within a given scan area can be adjusted by the camera operator. In other words, every OCT image acquisition produces a 3D image volume, composed of multiple B-scans, but the density of the B-scans (distance between each cross-sectional image) varies depending on the scanning protocol. In retina clinical practice and imaging research involving OCT images, each image acquisition (3D volume) is accessed and usually viewed one B-scan at a time, but the B-scans typically are not aligned. The misalignment of the B-scans does not pose a challenge for clinicians, who are primarily interested in the presence or absence of pathologies in individual B-scans, but this lack of alignment of B-scans could preclude analysis of OCT images en bloc as a 3D volume for machine learning purposes.

Being able to analyze OCT images en bloc as a 3D volume has significant implications for deep learning (DL) neural network-based image classification. Within the context of DL-based image classification, most published DL studies on macular OCT only utilized individual 2D B-scans as units of data input. Examples include differentiating between normal vs. AMD [8] and differentiating between choroidal neovascularization vs. diabetic macular edema vs. drusen vs. normal [9]. While choosing 2D B-scans within a 3D OCT volume as units of data for training and testing simplifies data curation and side-steps the complexity of 3D neural networks, this is not ideal, as only a portion of the available information is utilized. A more advanced strategy involves analyzing the entire 3D OCT volume en bloc. However, this will require efficient and accurate alignment of the B-scans within the same OCT 3D volume in the first place, and a tool that can achieve this is not readily available to investigators interested in 3D OCT imaging research.

### Image alignment

Image alignment [10–12] is a method of optimally registering one or more images onto a target image. Image alignment has many practical applications in medical image processing and analysis [13–15]. Medical image alignment can be divided into intra-subject (images from the same patient), inter-subject (from different patients) and atlas alignment (alignment of patient data to an atlas). To align the B-scans within the same OCT volume (intra-subject), the spatial transformation may be either a rigid or non-rigid deformable transformation. In this paper, we focused on three-parameter (one rotation and two translations) rigid alignment, and evaluated five medical image alignment methods: Advanced Normalization Tools Symmetric image Normalization (ANTs-SyN) [16], FMRIB’s Linear Image Registration Tool (FLIRT) [17], Insight Toolkit (ITK) [18], Optimized Automatic Registration (OAR) [19], TOADS-CRUISE (TOADS) [20], with a cross-correlation cost function.

### Our work

For macular OCT images, there is currently no consensus on how to assess the correct alignment of individual B-scans within a 3D volume. To this end, we propose a novel metric, the en face surface smoothness, which is more specific to B-scan-to-B-scan signal change and corroborates with the actual anatomy of a human macula, i.e., it is medically accurate that the inner retinal surface of a human macula, in the absence of surgical manipulation or major trauma, is smooth. We aligned the OCT images from all patients in our dataset, using the five medical image alignment methods.

The key contributions of our paper are: (1) developed of a novel metric, the en face surface smoothness, to quantify OCT B-scan alignment that is based on the actual anatomy of human maculae, and (2) compared and contrasted five commonly-used and readily-available alignment algorithms, and identified the algorithm with the best performance to align OCT B-scans within a 3D volume on the current image samples. To further demonstrate the utility of OCT B-scan alignment, we trained a 3D convolutional neural network (CNN) to detect age-related macular degeneration (AMD) on OCT images and compared the performance of the model with and without B-scan alignment. We chose to use AMD as a use case, as AMD is a leading cause of blindness in the world and OCT is indispensable in the diagnosis and management of AMD.

## Methods

### Dataset

The dataset [21] used in the alignment experiments was acquired at the Noor Eye Hospital in Tehran and consists of 50 normal and 48 non-neovascular AMD OCT volumes, acquired with a spectral domain optical coherence tomography system [Spectralis, Heidelberg, Germany]. For this dataset, the axial resolution is 3.5 μm with a scan area of 8.9 × 7.4 mm^2^. The number of A-scans was either 512 or 768 and the number of B-scans per OCT volume varied from 19, 25, 31, to 61.

The dataset used in the classification experiment was acquired at Duke University (230 OCT volumes; 115 AMD; 115 normal control) [22]. The volumes were acquired in a scan area of 6.7 × 6.7 mm centered on the fovea with a rapid scan protocol, resulting in volumes of 1000 × 512 × 100 voxels. The data was randomly split into training (56%, N = 130), validation (22%, N = 50) and test (22%, N = 50) and was partitioned at the patient level.

### B-scan rigid alignment

We used five commonly used algorithms to align the B-scans in each 3D-OCT volume: ANTs, FLIRT, OAR, ITK and TOADS. Advanced Normalization Tools Symmetric image Normalization (ANTs-SyN) was proposed in 2007 by the University of Pennsylvania [23], which developed a novel symmetric diffeomorphic optimizer for maximizing the cross-correlation in the space of topology preserving maps. The FMRIB's Linear Image Registration Tool (FLIRT) alignment algorithm [10, 19, 24] was proposed in 2000 by the University of Oxford and investigated assumptions underlying the problem of aligning brain images using a cross-modal voxel similarity measure. The Insight ToolKit image alignment (ITK) [18] alignment algorithm was proposed in 2003 by the University of Pennsylvania. Optimized automatic alignment 3D (OAR) [19] was a method proposed for optimal alignment based on FLIRT. The OAR technique specifies a transformation that minimizes a cost function, which represents the quality of alignment between two images. The TOADS-CRUISE Brain Segmentation Tools (TOADS) [25] is a series of software plug-ins developed by Johns Hopkins University in 2009 for automatic segmentation of magnetic resonance brain images, which was then adopted for OCT alignment with deformable registration.

We use $\left\{{R}_{1},{R}_{2},{R}_{3},...{,R}_{M-1},{R}_{M}\right\}$ to represent the B-scan images of a particular patient and R_M/2_ is the central (foveal) B-scan image. The B-scan alignment starts at the center (fovea) and so R_M/2–1_ and R_M1/2+1_ were aligned to R_M/2_. The alignment was propagated outward from the center and is outlined in Algorithm 1.
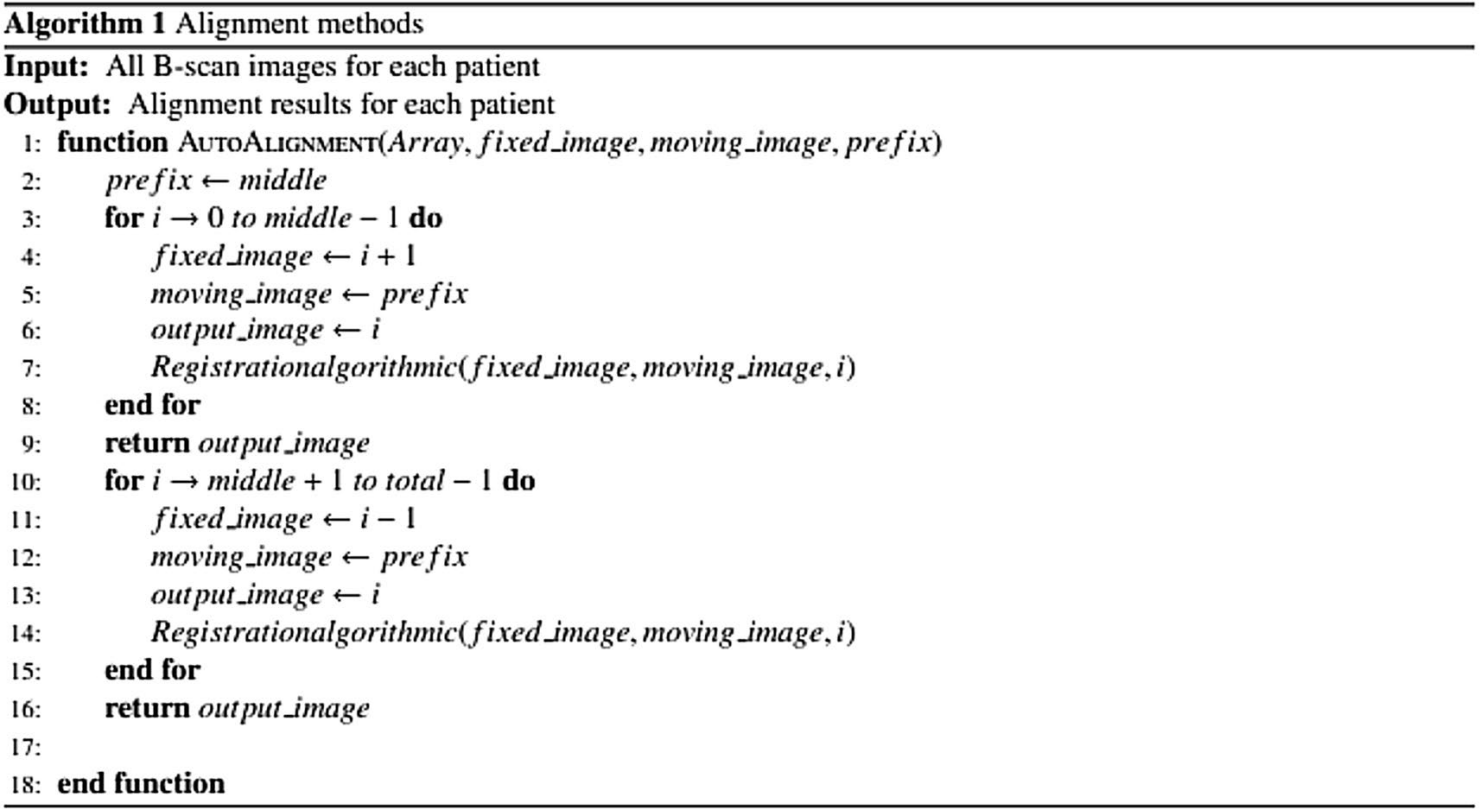


### Building the surface map and edge map

**Fig. 1:**
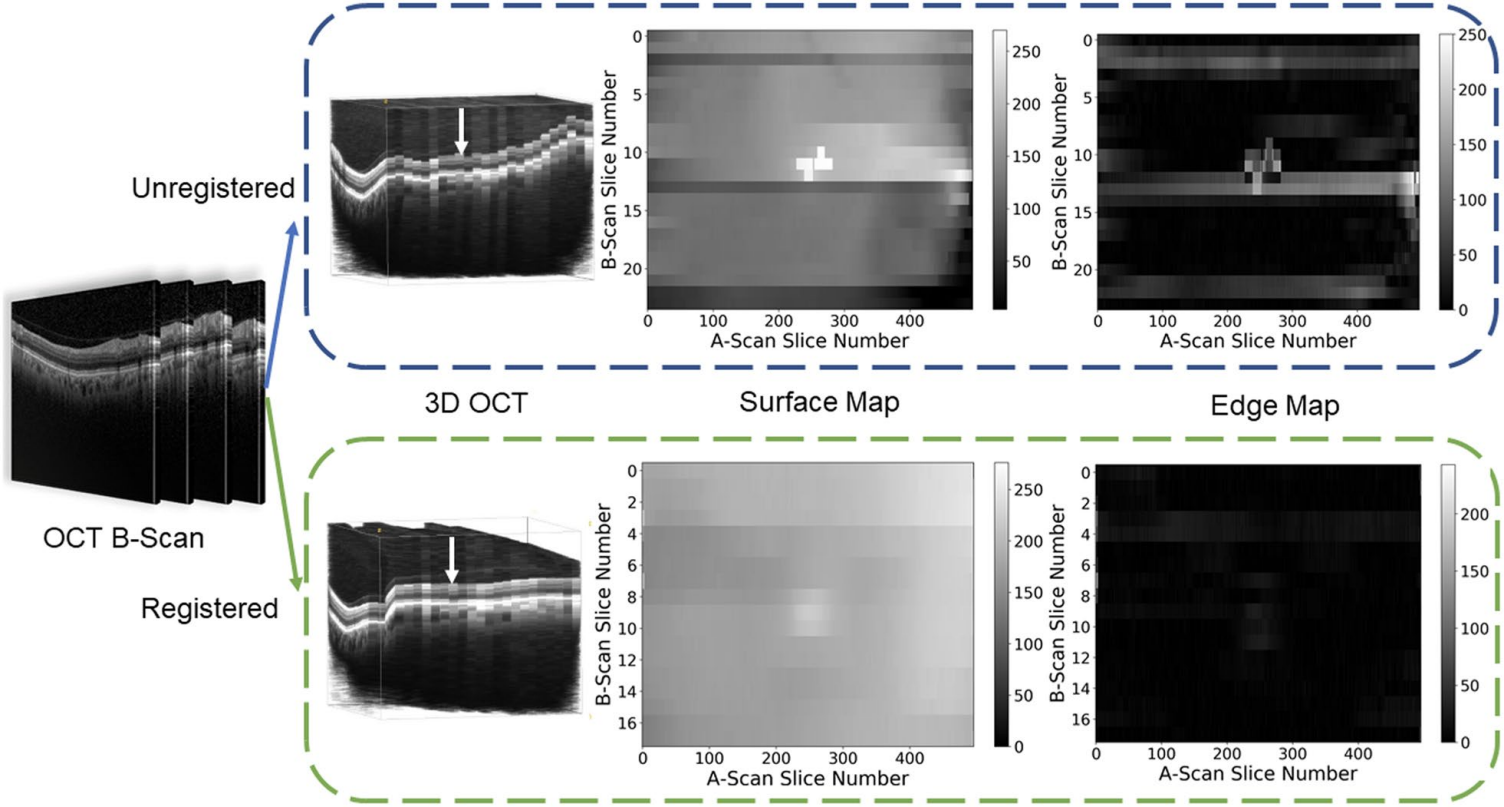
Multiple OCT B-scans from the same patient scan (far left) are combined to form a 3D cube. The surface map is defined as the distance from the top of the data cube to the top of the nerve fiber layer (white arrow). It is expected that well aligned B-scans will result in a smooth surface map. The measure of the smoothness is defined as the mean value of the difference map which is created by applying a discrete Laplace operator over the surface map

After the B-scans were combined (aligned or not aligned) into a 3D volume, an en face surface map was created. Figure 1 is a schematic representation for how the surface smoothness metric was generated. Multiple OCT B-scans from the same volume (eye) scan were combined to form a 3D cube. The surface map was defined by the distance from the top of the data cube to the top of the nerve fiber layer (Fig. 1, white arrow). We automatically remove the background noise from each B-scan image by the mean-shift [26] clustering algorithm to locate the nerve fiber layer of each B-scan image. The retinal surface located by the clustering algorithm was subsequently fit using a third order spline with locally weighted smoothing [27] (statsmodel.nonparametric.lowess from https://www.statsmodels.org) to remove very small jagged edge artifacts from the result of the clustering algorithm. Subsequently, the surface map was created by finding the nerve fiber layer of each B-scan. It is expected that well aligned B-scans will result in a smooth surface map (Fig. 1, bottom row) compared to unaligned B-scans (Fig. 1, top row). The measure of the smoothness is defined as the mean value of the edge map, which is created by applying a discrete Laplace operator over the surface map.

The edge map was calculated by applying Laplace edge detection algorithm [28] to the surface map. The Laplace operator is the simplest isotropic differential operator that is rotationally invariant and is defined as:

$$Laplace(f)=\frac{{\partial }^{2}f}{\partial {x}^{2}}+\frac{{\partial }^{2}f}{\partial {y}^{2}}$$

The mean of the edge map was automatically calculated and used as the measure of the smoothness of the surface map.

### Surface map validation

To validate the algorithm for generating the surface map, we generated two sets of surface maps for six patients (three AMD and three control patients; 360 B-scan images in total). The first set was generated by manual annotation of the nerve fiber layer (the surface layer of the retina). The second set was generated automatically using the method described above. The difference between the two sets of surface maps was represented by a histogram.

### Neural network model and training

We used the C3D CNN as the backbone of our model [29]. The training parameters included 2000 epochs, learning rate of 0.0001 (learning rate is divided by 10 every 50 epochs), Adam optimizer, cross entropy loss function, and a batch size of 2. The OCT signal was normalized to zero mean and scaled to unit standard deviation. Image augmentation consisted of a random horizontal flip. The proposed method was implemented using the Pytorch framework in Python with NVIDIA Cuda v10.0 and cuDNN v7.2 acceleration libraries. All experiments were performed on a Windows 10 machine with an Intel Core i7-7700 K 3.60 GHz CPU and an NVIDIA RTX TITAN GPU with 32 GB memory.

### Statistical method

We expected the smoothness of the surface map to increase, and therefore the difference in the surface map to decrease, after B-scan alignment. The mean difference in surface map smoothness before and after alignment was compared, using paired-sample t-tests with the null hypothesis that the two means were the same.

## Results

### Qualitative results

**Fig. 2:**
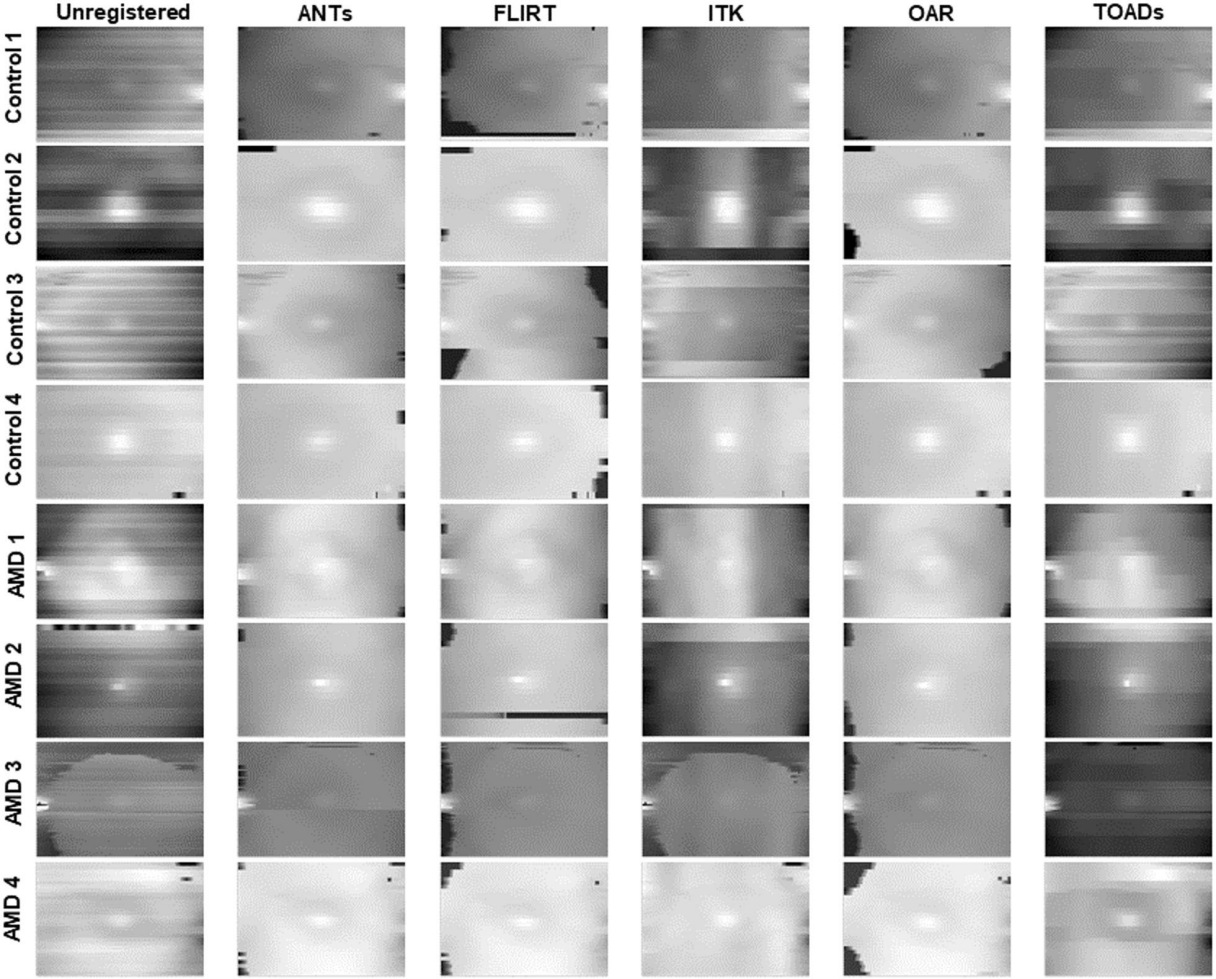
En face surface maps of OCT volumes. Each row represented a different patient, and each column represented a different alignment algorithm. The left most column was created from unregistered B-scans. (*AMD* Age-related Macular Degeneration)

Figure 2 was created to demonstrate visually the kind of artifacts that could be introduced if no B-scan alignment is performed, and shows the surface maps from four sample control and four sample AMD OCT volumes. Each row represents a separate eye. Each column represents a different alignment algorithm: ANTs [16, 30, 31], FLIRT [32–34], ITK [35–37], OAR [38] and TOADS [39, 40]. The left-hand column shows the en face surface map using unregistered B-scans. In general, the alignment algorithms created a smoother inner retinal surface for all eight eyes. In addition, these are our observations. First, the horizontal streaks in the images (e.g. Control 2 and AMD 1) represented significant mis-alignment of the B-scans along the Z axis in the OCT volumes. Second, the signal intensity differences seen in Control 3 and AMD 3 were due to mis-alignment between the central and peripheral regions within the area of the macula that underwent imaging. Third, within the same eye, such as Control 3, there were significant variations in performance between the algorithms. Fourth, black points that were typically seen near the edges were artifacts created by incorrect surface depth estimates (and quantified as “edge errors” in Fig. 4). Of the five algorithms, the ANTs alignment algorithm appeared to have both the highest degree of surface smoothness and fewest edge errors.

### Quantitative results

In this section, the alignment results will be compared quantitatively. The inner retinal surface of a human macula, in the absence of surgical manipulation or significant trauma, should be smooth. Thus, the success of the alignment algorithms was quantified by the mean of the Laplace difference across an en face surface map. The smaller the mean of the Laplace difference, the smoother the surface map, and the better the alignment.

**Fig. 3:**
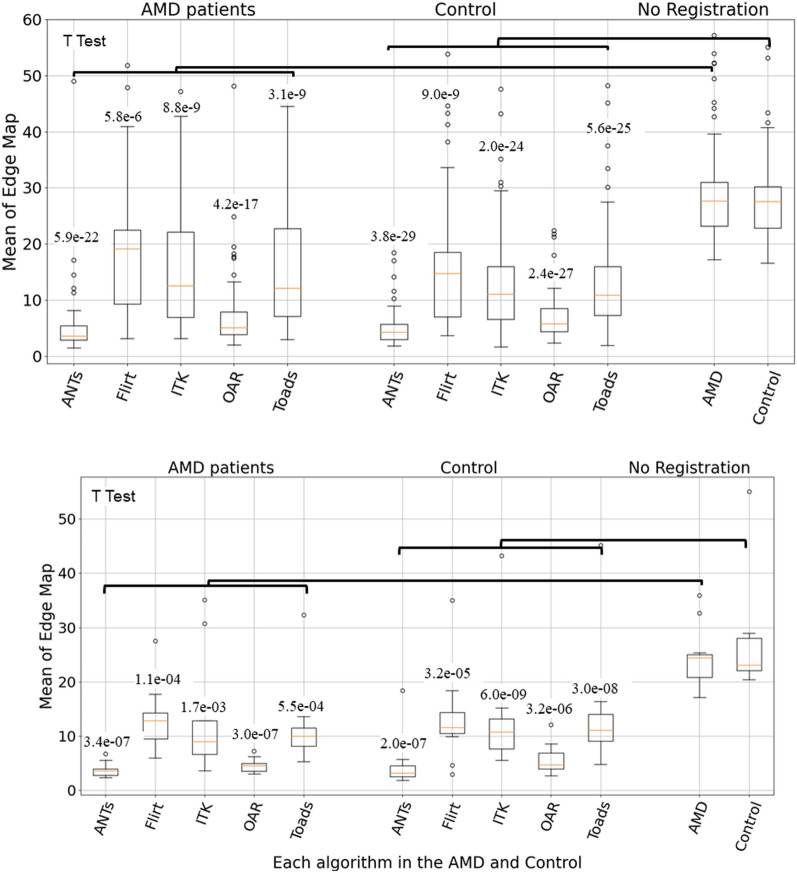
Mean of the Laplace difference of the surface map over all OCT volumes for each of the registration algorithms for control and AMD patients (top). The bottom panel only included OCT volumes with 61 B-Scans. Number above each box is the p-value from a paired t-test. (The circles in the figure are outliers and are discussed at the end of the paper)

When all OCT volumes were included for analysis, the mean Laplace difference for AMD and Control groups without alignment was 27.0 ± 4.2 pixels and 26.6 ± 4.0 pixels, respectively (Fig. 3, top panel, right). Within the AMD group, ANTs and OAR performed the best, with a mean Laplace difference of 5.5 ± 2.7 pixels and 8.1 ± 4.3 pixels, respectively. The mean Laplace difference for the FLIRT, ITK and TOADS algorithm was 16.2 ± 7.4 pixels, 14.7 ± 8.0 pixels, and 15.3 ± 8.5 pixels, respectively. For the AMD group, the mean Laplace difference for all five algorithms was statistically smaller than without registration (p < 0.05). Within the control group, ANTs, and OAR performed the best, with a mean Laplace difference of 4.3 ± 1.4 pixels and 6.5 ± 2.2 pixels, respectively. The mean Laplace difference for the FLIRT, ITK and TOADS algorithm was 12.9 ± 6.1 pixels, 11.8 ± 5.3 pixels, and 11.8 ± 4.7 pixels, respectively. Similarly for the control group, the mean Laplace difference for all five algorithms was statistically smaller than without registration (p < 0.05).

The data set used in this paper contained OCT volumes with varying B-scans densities, from 19 to 61 line scans. The higher the number of B-scans within an OCT volume, the more information it contains. Hence, we performed the same quantitative analysis, as above, to validate our approach specifically for high-density OCT images (OCT volumes with 61 B-scans) that are typically used in clinical practice or research (Fig. 3, bottom panel).

When only OCT volumes with 61 B-scans were included for analysis, the mean Laplace difference for the AMD and control groups without alignment was 23.1 ± 2.4 pixels and 25.1 ± 3.2 pixels, respectively. Within the AMD group, ANTs and OAR performed the best, with a mean Laplace difference of 3.5 ± 0.8 pixels and 4.2 ± 0.9 pixels, respectively. The mean Laplace difference for the FLIRT, ITK and TOADs algorithm was 11.7 ± 3.8 pixels, 9.6 ± 3.3 pixels, and 9.2 ± 2.3 pixels, respectively. For the AMD group, the mean Laplace difference for ANTs, OAR and TOADS was statistically smaller than without registration (p < 0.05). Within the control group, ANTs and OAR performed the best, with a mean Laplace difference of 4.0 ± 1.5 pixels and 6.0 ± 2.4 pixels, respectively. The mean Laplace difference for the FLIRT, ITK and TOADS algorithm was 12.4 ± 2.5 pixels, 9.6 ± 3.9 pixels, and 11.7 ± 2.7 pixels, respectively. For the control group, the mean Laplace difference for all five algorithms was statistically smaller than without registration (p < 0.05).

**Fig. 4:**
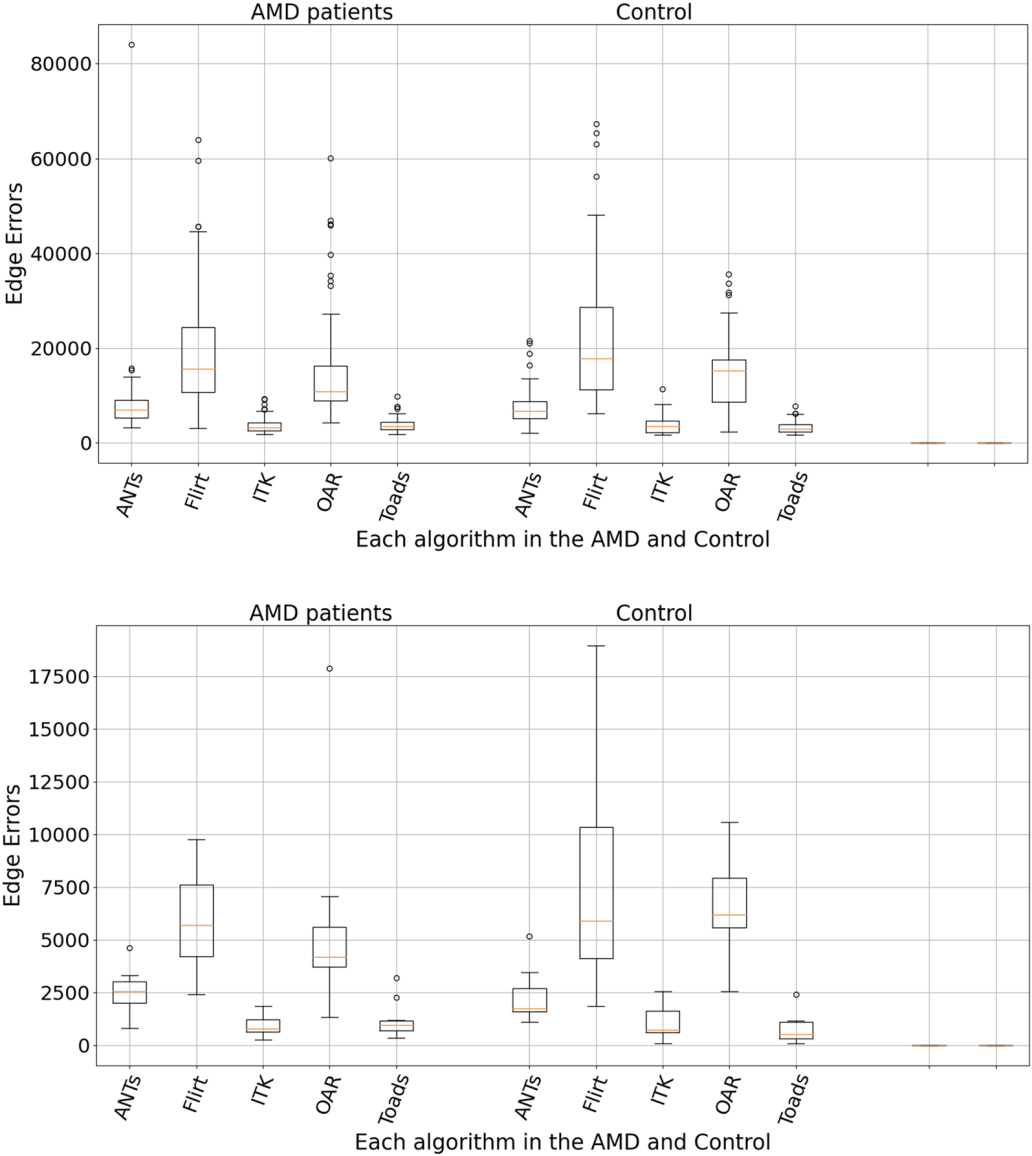
Mean number of edge errors over all OCT volumes for each of the registration algorithms for control and AMD patients (top). The bottom panel only included OCT volumes with 61 B-Scans

Figure 4 shows the edge errors for each algorithm for both the AMD and control groups. The top panel of Fig. 4 shows the results when all OCT volumes were included. The bottom panel of Fig. 4 shows the results when only OCT volumes with 61 B-scans were included. Overall, TOADS performed the best. For TOADS, the mean number of edge errors was 3586 ± 812, 3075 ± 829, 848 ± 346, and 701 ± 418 for the AMD (all OCT volumes), control (all OCT volumes), AMD (OCT with 61 B-scans only) and control (OCT with 61 B-scans only) group, respectively.

### Surface map validation results

**Fig. 5:**
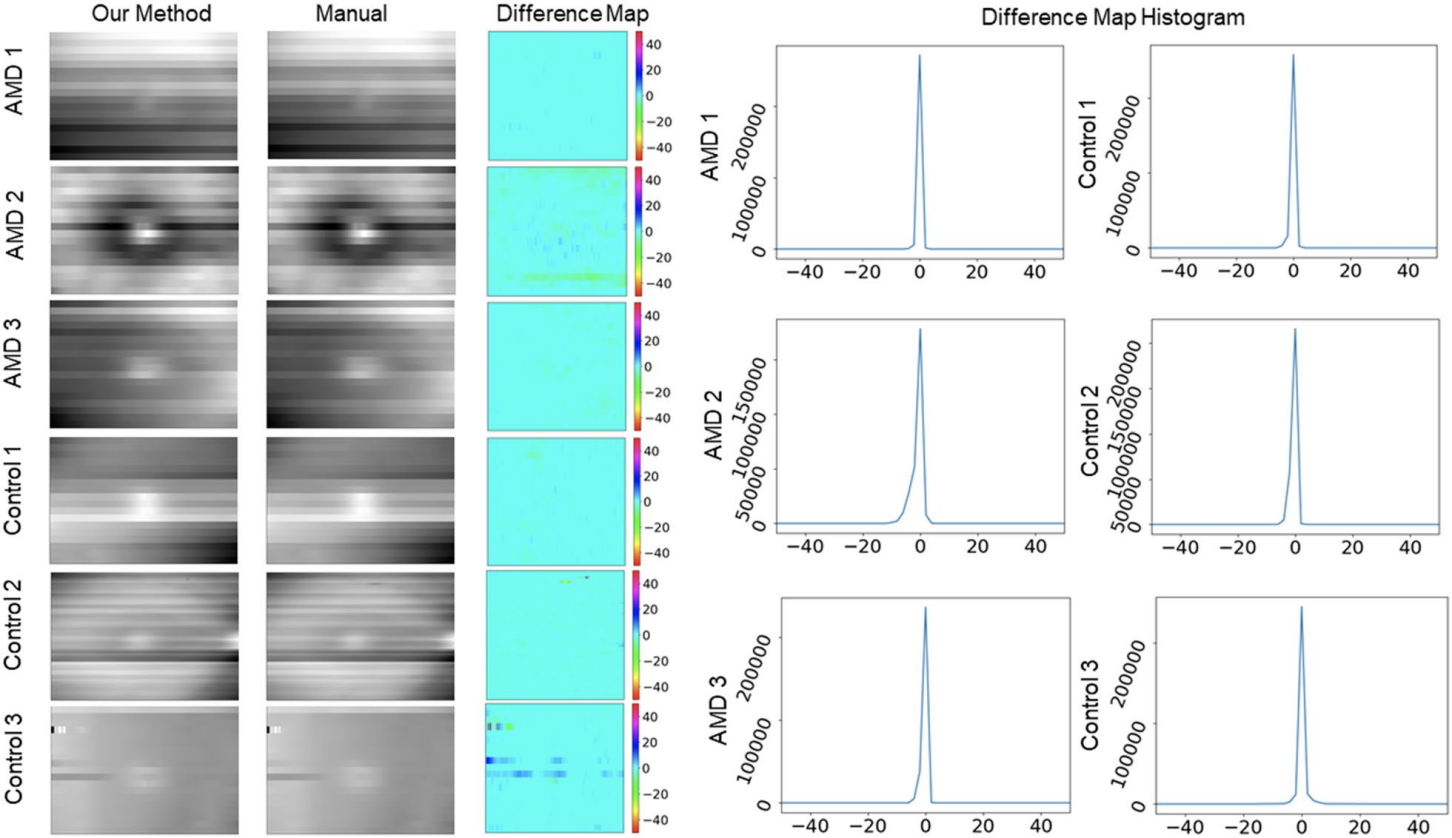
(Left) visualization and comparisons of the surface maps created manually and automatically. (Right) Histograms showing the difference between each pair of surface maps in the number of pixels

To validate the accuracy of our method for automatic surface map generation, we calculated the difference between manually generated surface maps and automatically generated surface maps, using B-scan images from 6 eyes. The difference was represented by a histogram and measured in pixels (Fig. 5). The mean and standard deviation in pixels of the difference between the six sets of surface maps was 0.28 ± 0.1, 1.4 ± 1.9, 0.45 ± 0.61, 0.5 ± 0.44, 0.99 ± 1.18, 1.21 ± 2.14, respectively.

### Algorithm speed

**Fig. 6:**
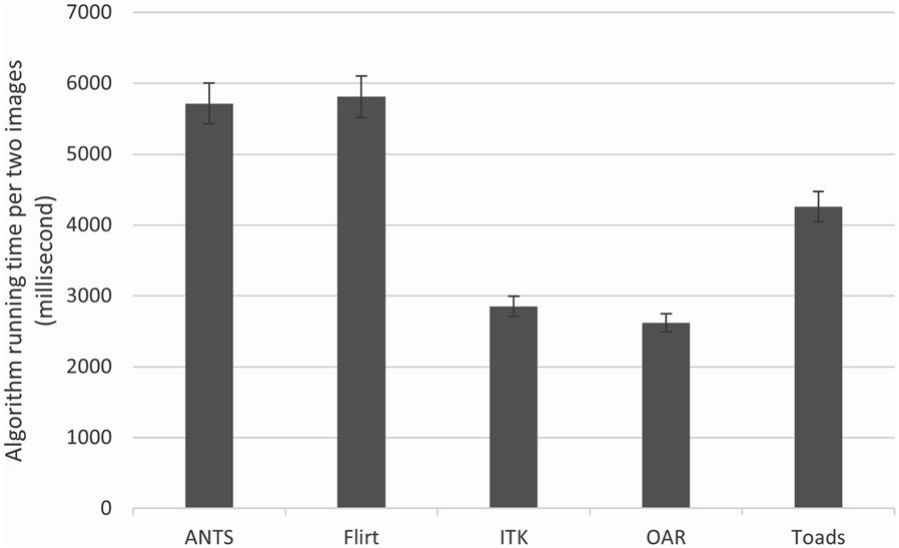
Average registration time in milliseconds per pair of B-scans for each algorithm

The performance was quantified by the mean time (in milliseconds) to register a pair of B-scans images over all B-scans within the same OCT volume (Fig. 6). The fastest algorithm was OAR at approximately 2500 ms per pair. The slowest algorithm was FLIRT at approximately 5,500 ms.

### Classification performance with and without alignment

We trained a 3D CNN to distinguish between normal and AMD OCT volumes. When our model was trained with B-scans aligned with ANTs, our model demonstrated superior performance (AUC 0.95 aligned vs. 0.89 unaligned; Table 1).

**Table 1:** Comparison of 3D CNN model performance with and without B-scan alignment in distinguishing between normal and AMD OCT volumes

	Sensitivity (95% confidence interval)	Specificity (95% confidence interval)	AUC (95% confidence interval)
Unaligned	0.88 (0.71, 0.92)	0.90 (0.73, 0.93)	0.89 (0.89, 0.94)
Aligned	0.93 (0.75, 0.94)	0.94 (0.76, 0.96)	0.95 (0.94, 0.98)

## Discussion

In this paper, we compared five commonly used alignment algorithms to register B-scans within an OCT volume, and tested our approach on a publicly-available dataset that contained both AMD and control patients. Overall, all five algorithms improved alignment, as measured by the mean Laplace difference of the en face surface map. Considering the results of all experiments, ANTs performed the best across all groups (AMD vs. control, all OCT volumes vs. OCT volumes with 61 B-scans only) in terms of producing the smoothest surface maps. In addition, we demonstrated that training a 3D CNN with B-scans aligned by ANTs produced a more robust model in detecting AMD on OCT volumes, as compared to training with unaligned B-scans.

Although ANTs did not produce the smallest number of edge errors, the proportion of edge errors as a mean percentage of total number pixels was still low at 0.04%. Hence, we believe that of the commonly available image alignment algorithms, ANTs should be used to register non-aligned B-scans within the same OCT volume. In image alignment, there are two options: rigid body transformation and nonlinear transformation [41]. For this paper, we only used rigid alignment methods, as many macular diseases, such as neovascular age-related macular degeneration and diabetic macular edema, manifest as perturbation of the retinal laminations on OCT imaging. We specifically avoided using non-linear warping techniques, as non-linear warping could inadvertently produce perturbation of the retinal layers and introduce artifactual pathologic features.

The number of B-scans within the OCT volumes varied from 19 to 61 for the dataset we used. That is, with the same scan area, the number of cross-sectional images varied from 19 to 61, and the distance between individual B-scans was inversely related to the number (density) of B-scans. The strength of our paper lies in the validation of our approaches using OCT volumes with different B-scan densities, as in real-world clinical practice, the B-scan density is often variable and determined by operator or scanning protocol preferences. In addition, the algorithms investigated in our study are already readily available to the public, so our findings are relevant to the broader research community.

Our manuscript was motivated by the fact that when OCT volumes are exported from commercially-available viewing software, an OCT volume is typically not exported en bloc but as a series of separate B-scans. However, 3D CNNs, one of the cutting-edge model architectures, require training data input in the form of 3D image volumes. Meaning, after the initial data export, the separate OCT B-scans will have to be re-combined into a 3D volume, before a 3D CNN can be trained. However, in the process of re-combining the B-scans, artifacts could be introduced if B-scan alignment is not performed. For example, if the retinal pigment epithelium (RPE) is not aligned between adjacent B-scans, an artifactual “RPE break” could be introduced. To demonstrate the utility of aligning OCT B-scans, specifically within the context of training 3D CNN models for DL-based image analysis, we trained a model to detect AMD in 3D OCT volumes. We compared the performance of our model, with and without B-scan alignment, and demonstrated that B-scan alignment produced a more robust model. Our data provides proof-of-concept evidence that aligning B-scans could be broadly useful for training any 3D CNNs that involve 3D macular OCT volumes.

## Limitations

Our paper has several limitations. First, none of the five algorithms included in this study were originally developed for OCT B-scan registration, so an algorithm designed from the ground up for this specific purpose may yield even better results. Second, the range of pathologies included in this OCT dataset was limited, as only control (normal) and AMD patients were included. Specifically, drusen, defining features of AMD, are located in the outer retina and do not significantly perturb the smoothness of the inner retinal surface. For conditions that introduce significant inner retinal surface perturbation, such as trauma and localized irregular epiretinal membrane, our novel metric of surface smoothness may not be applicable.

## Conclusions

In this paper, we introduced a novel metric to quantify OCT B-scan alignment and compared the effectiveness of five alignment algorithms. We confirmed that alignment could be improved in a statistically significant manner with readily available alignment algorithms that are available to the public, and the ANTs algorithm provided the most robust performance overall.

## Data Availability

The datasets used and/or analysed during the current study are available from the corresponding author on reasonable request.

## Abbreviations

OCT: Optical coherence tomography
AMD: Age-related macular degeneration
ANTs-SyN: Advanced normalization tools symmetric image normalization
MRI: Magnetic resonance imaging
DL: Deep learning;
FLIRT: FMRIB’s linear image registration tool
ITK: Insight toolkit
OAR: Optimized automatic registration
TOADS: TOADS-CRUISE
